# Spatially and temporally distinct patterns of expression for VPS10P domain receptors in human cerebral organoids

**DOI:** 10.3389/fcell.2023.1229584

**Published:** 2023-09-29

**Authors:** Fabia Febbraro, Helena Hørdum Breum Andersen, Meagan M. Kitt, Thomas E. Willnow

**Affiliations:** ^1^ Department of Biomedicine, Aarhus University, Aarhus, Denmark; ^2^ Max Delbrueck Center for Molecular Medicine, Charité—Universitätsmedizin Berlin, Berlin, Germany; ^3^ Max Delbrueck Center for Molecular Medicine, Berlin, Germany

**Keywords:** Alzheimer’s disease, cerebral organoids, neuronal protein sorting, SORLA, sortilin, SORCS

## Abstract

Vacuolar protein sorting 10 protein (VPS10P) domain receptors are a unique class of intracellular sorting receptors that emerge as major risk factors associated with psychiatric and neurodegenerative diseases, including bipolar disorders, autism, schizophrenia, as well as Alzheimer’s disease and frontotemporal dementia. Yet, the lack of suitable experimental models to study receptor functions in the human brain has hampered elucidation of receptor actions in brain disease. Here, we have adapted protocols using human cerebral organoids to the detailed characterization of VPS10P domain receptor expression during neural development and differentiation, including single-cell RNA sequencing. Our studies uncovered spatial and temporal patterns of expression unique to individual receptor species in the human brain. While *SORL1* expression is abundant in stem cells and *SORCS1* peaks in neural progenitors at onset of neurogenesis, *SORT1* and *SORCS2* show increasing expression with maturation of neuronal and non-neuronal cell types, arguing for distinct functions in development *versus* the adult brain. In neurons, subcellular localization also distinguishes between types of receptor species, either mainly localized to the cell soma (*SORL1* and *SORT1*) or also to neuronal projections (*SORCS1* and *SORCS2*), suggesting divergent functions in protein sorting between Golgi and the endo-lysosomal system or along axonal and dendritic tracks. Taken together, our findings provide an important resource on temporal, spatial, and subcellular patterns of VPS10P domain receptor expression in cerebral organoids for further elucidation of receptor (dys) functions causative of behavioral and cognitive defects of the human brain.

## Introduction

**FIGURE 1:**
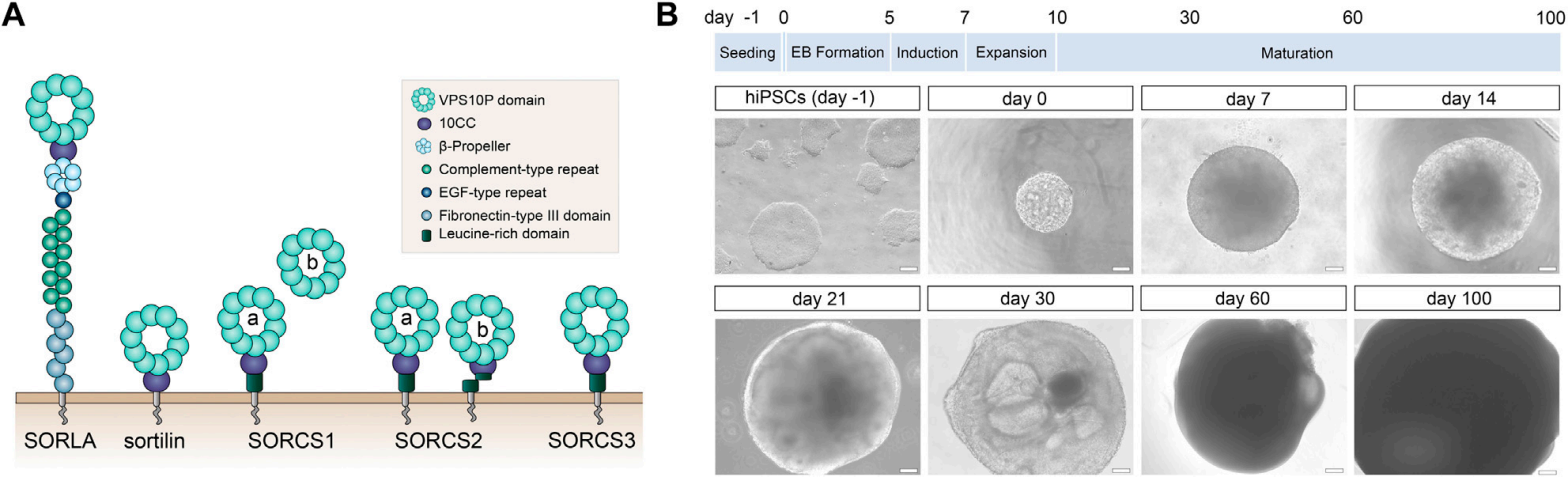
Human cerebral organoids as model system to study VPS10P domain receptors **(A)** Structural organization of the mammalian VPS10P domain receptors sorting protein-related receptor with A-type repeats (SORLA), sortilin, as well as sortilin-related receptors CNS expressed (SORCS) -1, -2, and -3. These type-1 transmembrane proteins share a common vacuolar protein sorting 10 protein (VPS10P) domain that constitutes the main ligand binding site. Their ectodomains may harbor additional protein modules involved in protein-protein interactions. Their cytoplasmic tails contain binding motifs for sorting adaptors directing trafficking of the receptors between cell surface and intracellular compartments. For reasons of simplicity, all receptors are depicted as monomers. However, active forms of SORCS1, -2, -3 likely constitute homodimers, whereas sortilin transiently dimerizes in endosomes to discharge its ligands. Active SORCS1 exists as membrane-bound full-length receptor (version a) and as secreted ectodomain (version b). SORCS2 is found as single- (version a) and as proteolytically cleaved two-chain variants (version b) that each carry distinct receptor functions. **(B)** The timeline for differentiation of human induced pluripotent stem cells (hiPSCs) into cerebral organoids is given. The appearance of exemplary organoids at the indicated timepoints is shown in brightfield images below. EB, embryoid body. Scale bars: 100 μm or 200 µm (for day 60 and 100).

Vacuolar protein sorting 10 protein (VPS10P) domain receptors are a specialized class of type-1 transmembrane receptors that sort target proteins between cell surface and intracellular compartments, defining endocytic and secretory capacities of cells. Mammalian members of the gene family encompass the sorting protein-related receptor with A-type repeats (SORLA), sortilin, as well as sortilin-related receptors CNS expressed (SORCS)-1, -2, and -3 (Figure 1A) Hermey (2009); Malik and Willnow (2020). In line with prominent expression of VPS10P domain receptors in the central and peripheral nervous systems, these receptors have been implicated in multiple psychiatric and neurodegenerative disorders. Among others, SORCS1, -2 and -3 have been identified as risk genes for bipolar disorder, attention deficit hyperactivity disorder (ADHD), autism, and schizophrenia (Baum et al., 2008; Christoforou et al., 2011; Lionel et al., 2011; Alemany et al., 2015). SORLA and sortilin are genetically associated with age-related neurodegeneration in Alzheimer’s disease (AD) (Rogaeva et al., 2007; Lambert et al., 2013; Bellenguez et al., 2022) and frontotemporal dementia (FTD) (Carrasquillo et al., 2010). The importance of VPS10P domain receptors as therapeutic targets for brain disorders is underscored by clinical trials applying sortilin antagonists for the treatment of FTD (https://www.alzforum.org/therapeutics/latozinemab).

Some immunohistological data are available on the expression of VSP10P domain receptors in the adult human brain, including SORLA and sortilin in neurons of frontal cortex and hippocampus (Scherzer et al., 2004; Offe et al., 2006; Xu et al., 2019), or SORCS2 in cortical neurons and astrocytes (Malik et al., 2020). Still, much of our knowledge about the expression and functional significance of these receptors in health and disease stems from studies in transgenic mouse models (Malik and Willnow, 2020; Salasova et al., 2022). While these studies have been instrumental in identifying fundamental processes controlled by these receptors, they also highlighted important differences in receptor biology in humans *versus* rodent models. For example, sortilin has been shown to promote lipoprotein particle secretion from mouse (Kjolby et al., 2010; Strong et al., 2012) but to block release from human cells (Musunuru et al., 2010). The role of SORLA as risk factor for both familial and sporadic forms of AD may be related to its expression in human microglia (Nott et al., 2019), yet relatively little expression in this cell type is seen in mice (Gosselin et al., 2017; Hansen et al., 2018). Obviously, validating the significance of single nucleotide polymorphisms (SNPs) associating VPS10P domain receptor genes with brain diseases also requires human cell models derived from carriers of such risk gene variants.

Towards validating human iPSC-derived models for the study of VPS10P domain receptors in development and function of the human brain, we carried out a detailed analysis of the spatial and temporal aspects of receptor expression in human cerebral organoids.

## Materials and methods

### Cell lines

The induced pluripotent stem cell (iPSC) line HMGUi001 was used in this study (https://www.ebi.ac.uk/biosamples/samples/SAMEA5696688). It was reprogrammed from fibroblasts obtained from a Caucasian female donor. The cells were grown in Essential 8 Flex Basal Media (Gibco, A28583-01, Thermofisher) on vitronectin-coated plates (07180 Stemcell technologies; NUNC DISH 35 mm, 150255, Thermoscientific). The cells were passaged every 3–4 days at a ratio of 1:10 using 0.5 mM EDTA in PBS for 4 min at room temperature.

### Generation of cortical organoids

For generation of cerebral organoids, we adapted published protocols (Lancaster et al., 2013; Giandomenico et al., 2021). On day −1, iPSCs were resuspended in StemFlex media containing 10 µM Rock inhibitor Y-27632 (Cayman Chemical Company, Bionordika) and 2000 cells in 150 µL of media were plated in each well of 96-well ultra-low attachment plates (174929, Thermofisher). Cells were centrifuged at 100 *g* for 3 min and incubated at 37°C (in 5% CO_2_) for 24 h. On day 0, 70 µL of media were removed from each well and replaced by 100 µL embryoid body (EB) formation medium (Cerebral organoid kit, cat. n. 08570, StemCell Technologies). On day 2, again 100 µL media were removed and the same amount of fresh EB formation media added back to each well. On day 5, 100 µL of media containing an organoid were removed from each organoid-containing 96-well using a pipette with 200 µL pipette tip. Once the residing organoids descended to the opening of the pipette tip by gravity, they were collected and moved to a new 96-well plate containing 150 µL of induction media (Cerebral organoid kit, cat. n. 08570, StemCell Technologies) per well. Special care was taken to only release the organoids from the tip by gravity and to exclude the contaminating EB medium. On day 7, 70 µL of media were removed from each well and replaced with 150 µL expansion medium (Cerebral organoid kit, cat. n. 08570, StemCell Technologies). On day 10, organoids were embedded in matrigel (Matrigel 356234 for organoids, BD Bioscience) as follows. Matrigel was thawed and kept on ice for at least 2 h before application to prevent polymerization. Using a 1 mL pipette tip, 32 organoids were collected from 96-well plates and placed in a 1.5 mL reaction tube. Once the organoids sunk to the bottom of the tube, the supernatant was removed and 60 µL of maturation medium were added, followed by 100 µL of Matrigel. The solutions were mixed gently, being careful not to stir up the organoids. Using a 200 µL pipette tip, the medium containing the organoids was recovered and plated in drops in ultra-low attachment 6-well plates (83.3920500, Starstedt). The plate was incubated at 37°C for 17 min, after which 4 mL maturation medium (Cerebral organoid kit, cat. n. 08570, StemCell Technologies) were gently added to each well. After 24 h incubation, EB were detached from the bottom of the wells using a 1 mL pipette tip and the 6-well plate placed back into the incubator on an orbital shaker set to 74 rpm. Penicillin-Streptomycin solution (0.5%, Cat No. 15140122, Life and Technologies) was added for cell culture and differentiation. The medium was changed every other day and organoids were collected at different time points for further analysis.

### Single-cell sample preparation

For 21 days old organoids, nine organoids were collected and placed in a 1.5 mL tube. The samples were washed briefly in PBS, before adding 200 μL of TrypLE Express solution (12604-013 Gibco). The samples were mixed by gentle pipetting and incubated at 37°C for 5 min. Then, the samples were manually dissociated by further pipetting and incubation for additional 5 min. This manual dissociation step was repeated three times. Thereafter, the dissociation reaction was stopped by addition of 800 μL of E8 Stem Flex medium and the cells were counted an automated cell counter (Countess II, Invitrogen) using the viability dye Trypan blue (0.4%, T10282, Invitrogen). To obtain a single cell preparation, the cell suspension was filtered through a 40 μm filter (22363547, Thermo fisher). Filtered cells were centrifuged for 2 min at 200 g, washed once with 1 mL of PBS containing 0.04% BSA (130-091-376, Miltenyibiotec), and resuspended by gentle pipetting to remove cell clumps. After this final step, cell viability was around 98%.

For 100 days old organoids, five samples were collected and placed in a 35 mm plate. To remove dead cells from the organoid core, organoids were cut in half using a syringe needle and washed with PBS. Subsequently, PBS was removed and 200 μL of TrypLE Express solution (12604-013 Gibco) was added. Next, the organoids were cut into smaller fragments using two needles attached to 1 mL syringes. Fragments were collected in a 1.5 mL reaction tube and subjected to gentle pipetting followed by intermittent resting at 37°C for 5 min. Single cells present in the supernatant after 5 min were collected into a new reaction tube and placed into the incubator. Tissue fragments remaining in the tube were treated with 100 μL of TrypLe Express and dispersed into small pieces using consecutive pipetting with 1 mL and 200 µL pipette tips. After additional 5 minutes, the single cells in the supernatant were again collected and added to the tube containing the single-cell suspension. This procedure was repeated 3 times for a total of 15 min. Any remaining tissue fragments were discarded. TrypLe Express dissociation was stopped by adding an equal volume of E8 Stem Flex medium, resulting in a total volume of 1 mL. Cell viability was assessed using the viability dye Trypan blue (0.4%, T10282, Invitrogen) and cells were counted using an automated cell counter (Countess II, Invitrogen). Thereafter, the single-cell suspension was filtered through a 40 μm filter (22363547, Thermo fisher) placed on top of a 50 mL tube. Filtered cells were collected and cell viability determined as above. Typically, cell viability at this step was around 50%–60%. To increase the percent of live cells in the single-cell suspension, the cells were centrifuged for 2 min at 200 g and placed on ice. The cell pellet was washed in 1 mL of PBS with 0.04% BSA (130-091-376, Miltenyibiotec), pipetted gently to remove any cell clumps, and counted again. This washing step was repeated two more times, resulting in a cell viability of approximately 80%.

### Single-cell RNA sequencing

Single cell suspensions were collected at a concentration of 1,000 cells/μL in PBS, 0.04% BSA for library preparation and sequencing. Libraries were generated using the Chromium Next GEM Single Cell 3ʹ Reagent Kits v3.1 Dual Index (10X Genomics Inc., United States). Details on numbers of cells loaded, recovered, and passing quality controls are given in Table 1. Briefly, a droplet emulsion targeting 8,000 cells was generated in a microfluidic Next GEM Chip G, followed by barcoded cDNA generation inside the droplets. Purified and amplified cDNA was then subjected to library preparation and sequenced on a NovaSeq 6,000 instrument (Illumina, United States) using a SP100 flow cell kit to a depth of 400M read-pairs per sample. Sequencing was performed as a service at the Department of Molecular Medicine (MOMA) of Aarhus University Hospital, Denmark (https://www.moma.dk/). Single-cell reference annotation was carried out by the NGS service provider Omiics (https://omiics.com). In brief, barcode processing and single-cell 3′gene quantification was done using the Cell Ranger Single-Cell Software Suite (v 3.1.0) using the GRCh38 reference genome. The Seurat R package (v 4.0.4) was used to quality filter cells and remove cells with above 20% of reads mapping to mitochondrial genes. Single cells were further filtered using the R package DoubletFinder (v.2.0.3) to remove the doublets. Integration of datasets was done in Seurat using reciprocal PCA (rPCA) integration. Furthermore, Seurat was used for data normalization, dimensionality reduction by use of the Uniform Manifold Approximation and Projection (UMAP) technique.

**TABLE 1:** Cell numbers in single-cell RNA sequencing.

	Cells loaded	Aim for recovery	Actual capture	QC filter: Mitochondrial genes <20% genes/cell >700	GC filter: Doublet removal
Day 21	8,000	5,000	5,200	4,858	4,761
Day 100	8,000	5,000	2,339	1,591	1,559

Clustering was done with Seurat’s graph-based clustering approach using the FindClusters function. Annotation of clusters was assigned manually based on previously published data of single-cell sequencing using cortical differentiation (Camp et al., 2015; Eze et al., 2021; Fleck et al., 2021; Ziffra et al., 2021). Marker genes were detected for each cluster using the Seurat function FindAllMarkers.

### Expression analyses

Transcript levels were determined by quantitative (q) RT-PCR. To do so, total RNA was extracted from organoids using RNeasy Mini Kit (70104, Qiagen) and reverse transcribed using High-Capacity RNA to cDNA kit (4387406, Thermo Fisher Scientific). Quantitative PCR reaction was performed using 5 ng of cDNA and the Taqman Fast Advanced Master Mix solution (4444557, Thermo Fisher Scientific). The samples were loaded on a MicroAmp EnduraPlate (4483285, Life and technologies) and run on the QuantStudio 7 Flex Real-Time PCR System instrument (Thermo Fisher Scientific). The probes used are given in Supplementary Table S1. Expression values were normalized to *GAPDH* as internal control.

### Organoid protein isolation and detection

Protein extracts were generated by homogenization of organoids in solubilization buffer (20 mM Tris, 2 mM MgCl_2_, 0.25 M sucrose, pH = 7,5) using sonication, followed by differential centrifugation at 10,000 x g (10 min, 4°C) and 100.000 x g (30 min, 4°C). The resulting supernatant was used to detect non-membrane bound soluble proteins. The protein pellet was resuspended in lysis buffer (20 mM Tris pH 8, 10 mM EDTA**,** 1% NP40**,** 1% Triton) containing complete protease inhibitor cocktail (Roche 11836145001) and Halt phosphatase inhibitor cocktail (Thermo Scientific 78427). Resuspension was carried out for 3 h on ice using repeated pipetting with a 30G syringe. This membrane fraction was used for detection of membrane-bound full-length VPS10P domain receptor species. Protein levels in membrane and soluble fractions were determined by standard Western blot analyses using primary and secondary antibodies given in Supplementary Tables S2, S3.

### Immunohistochemical analyses

Immunohistochemistry was performed on organoids fixed in 4% paraformaldehyde for 20 min, infiltrated in 20% sucrose over night at 4°C, and embedded in OCT compound (Tissue-Tek, Sakura) for standard 10 µm cryo-sectioning. For immunodetection, cryosections were washed with PBS for 10 min, blocked in 5% donkey serum (D9663, Sigma) in PBT (PBS with 0.25% Triton X-100) for 1 h at room temperature, followed by incubation with primary antibody in 2.5% donkey serum in PBT at 4°C overnight. Antibodies are listed in Supplementary Table S2. Next, tissue sections were washed 3x with PBS for 10 min, blocked with 1% donkey serum in PBST for 10 min, before treatment with secondary antibodies (1% donkey serum in PBT), for 1 h at room temperature. The secondary antibodies used are listed in the Supplementary Table S3. Finally, coverslips were washed in PBS and mounted on slides using Fluorescence Mounting Medium (S3023, Dako). Images were captured on a confocal microscope (Zeiss LSM800) using 10X and 63X lens.

### Statistical analysis

All experiments in this study encompass biological replicates from a minimum of 3 independent organoid differentiations. Statistical analysis was performed using GraphPad Prism 9. Data are given as the mean ± standard deviation (SD). Statistical significance of data was determined by one-way ANOVA followed by Dunnett’s multiple comparisons tests.

## Results

### VPS10P domain receptors show temporally distinct expression patterns during organoid development

To interrogate expression of VPS10P domain receptors during human brain development and in adult function, we modified published protocols for efficient and reproducible generation of cerebral organoids from human iPSC lines (see methods for details). Our protocol follows a time line of seeding of iPSCs, formation of embryoid bodies (EB), and subsequent maturation of the organoids for up to 100 days (Figure 1B). Cerebral organoids were chosen based on databases suggesting expression of all VPS10P domain receptors in the human brain cortex (https://www.proteinatlas.org).

**FIGURE 2:**
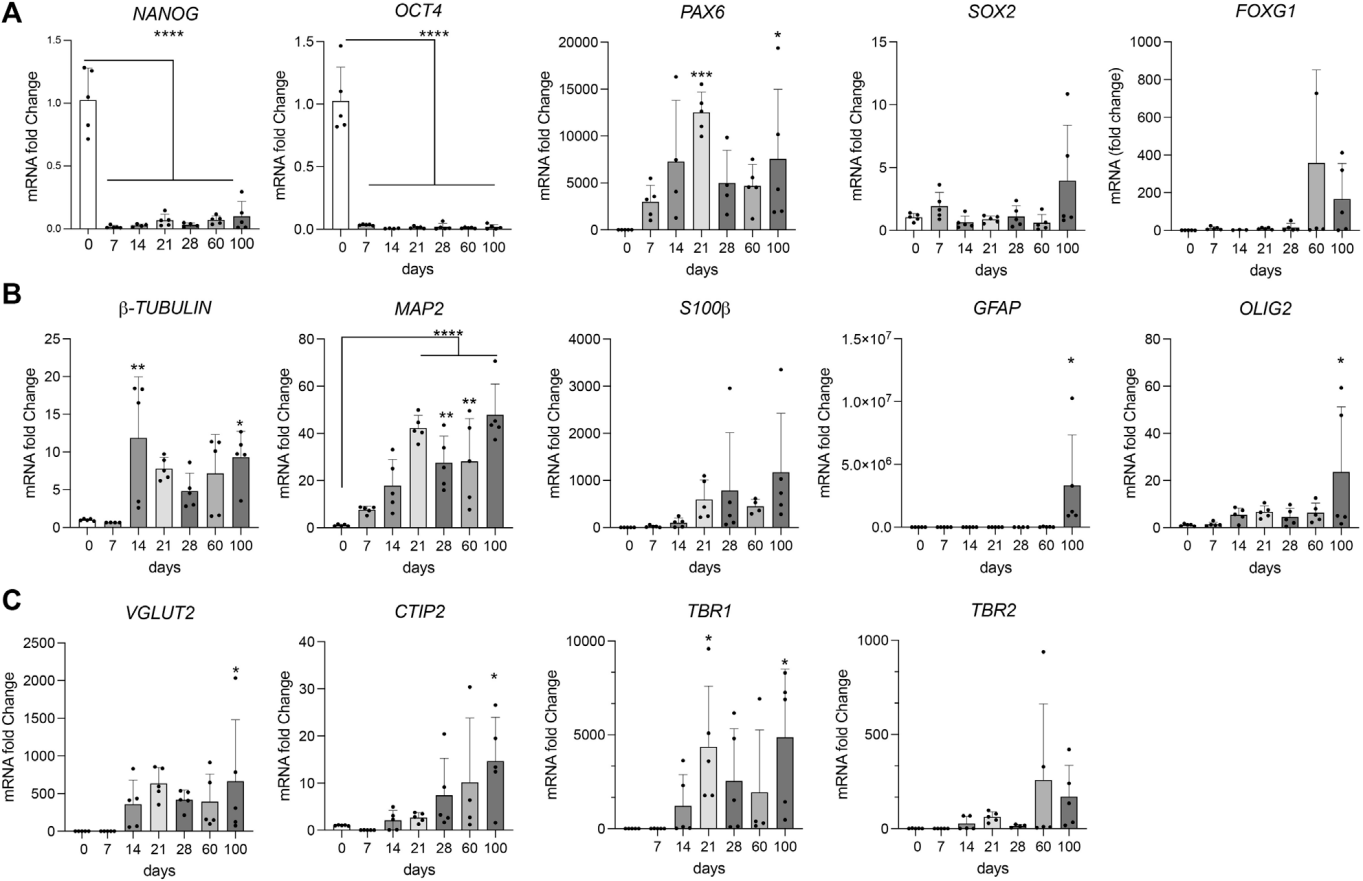
Marker gene transcription during organoid maturation Transcript levels for the indicated marker genes were determined by quantitative (q) RT-PCR in cerebral organoids at various timepoints of differentiation. In **(A)** stem cells and progenitor markers, in **(B)** mature neural markers; in **(C)** markers for different neuron subtypes. Data are given as changes relative to transcript levels in embryoid bodies (day 0 of differentiation set to 1). Statistical significance of data was determined by one-way ANOVA followed by Dunnett’s multiple comparisons tests. n = 3–5 organoids per condition (each organoid from an independent differentiation experiment); *, *p* < 0.05; **, *p* < 0.01; ***, *p* < 0.001; ****, *p* < 0.0001.

**FIGURE 3:**
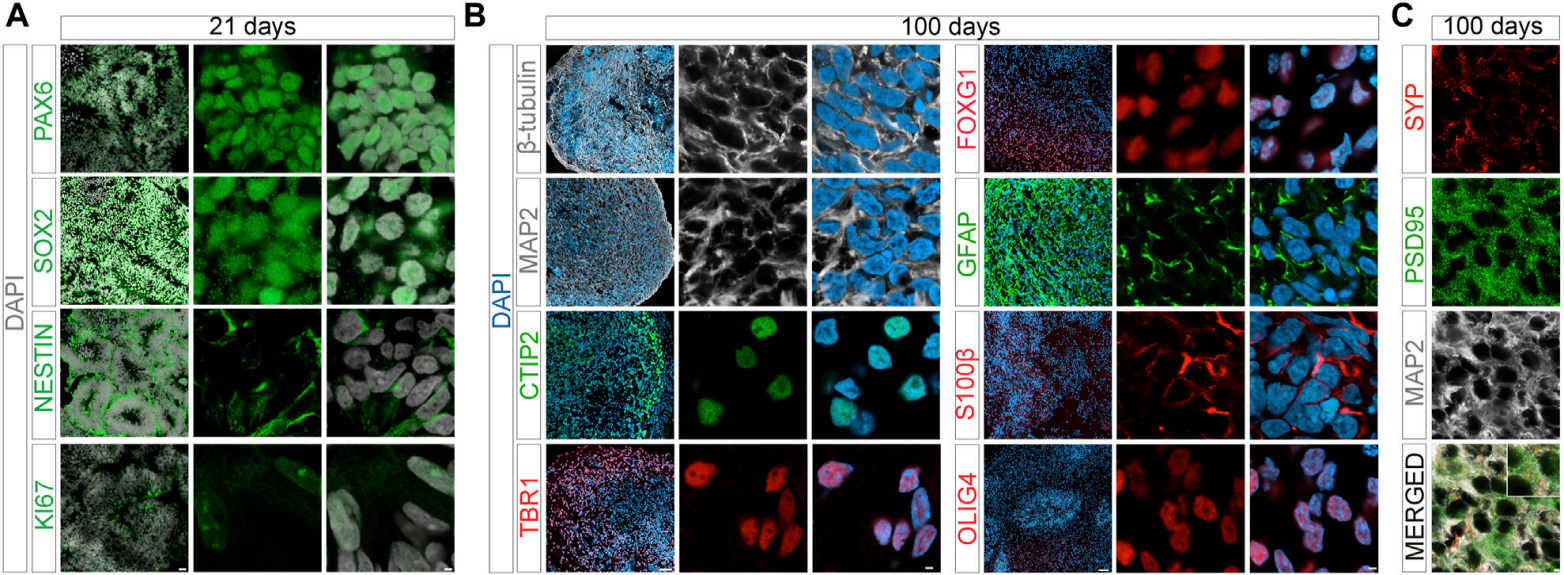
Marker protein expression in organoids at day 21 and day 100. Immunohistological analysis of expression of the indicated marker proteins in cerebral organoids at 21 **(A)** or 100 days **(B)** of differentiation. Overview images in the left panels show merged channel configurations. Higher magnification images to the right show marker expression without (middle panels) or with nuclear DAPI stain (right panels). **(C)** Co-immunostainings of pan-neuronal marker MAP2 with synaptophysin (SYP, pre-synapse) and post-synaptic density protein 95 (PSD95, post-synapse) in 100 days old organoids are shown. Images are given in single and merged channel configurations. The inset in the merged image highlights co-localization of MAP2 with both synaptic markers. Scale bars: 2 µm for high magnification images, 20 or 50 µm for overview images at day 21 and 100, respectively. CTIP2, COUP-TF-interacting protein 2; TBR1, T-Box Brain Transcription Factor 1.

During maturation, cerebral organoids showed the expected decrease in pluripotency markers *NANOG* and *OCT4*, and a corresponding induction of genes in early telencephalic development, including *PAX6* and *SOX2*, and an increase in the forebrain marker *FOXG1* (Figure 2A). From day 14 onwards, marker gene expression indicative of neural development, including pan-neuronal markers *TUBB* and *MAP2*, as well as neuroepithelial marker *S100β* was apparent. Expression of non-neuronal cell markers *GFAP* (astrocytes) and *OLIG2* (oligodendrocytes) was seen in mature organoids around 100 days of age (Figure 2B). Markers of cortical specification appeared around day 21, including *TBR2* (cortical neurogenesis), *VGLUT2* (glutamatergic neurons), as well as *CTIP2* and *TBR1* (cortical neurons layers V and VI) (Figure 2C). Induction of neurogenesis in proliferating progenitor populations was corroborated by immunohistological detection of lineage markers PAX6, SOX2, and nestin, as well as proliferation marker KI67 in day 21 organoids (Figure 3A). Subsequent formation of neuronal and non-neuronal cell types was confirmed by immunostaining for β-tubulin, MAP2, TBR1, CITP2, and FOXG1, as well as for GFAP, S100β, and OLIG4, respectively (Figure 3B). Colocalization of immunosignals for the pan-neuronal marker MAP2 with proteins indicative of pre- (synaptophysin, SYP) and post-synaptic (PSD95) compartments documented the formation of synapses by day 100 of organoid differentiation.

**FIGURE 4:**
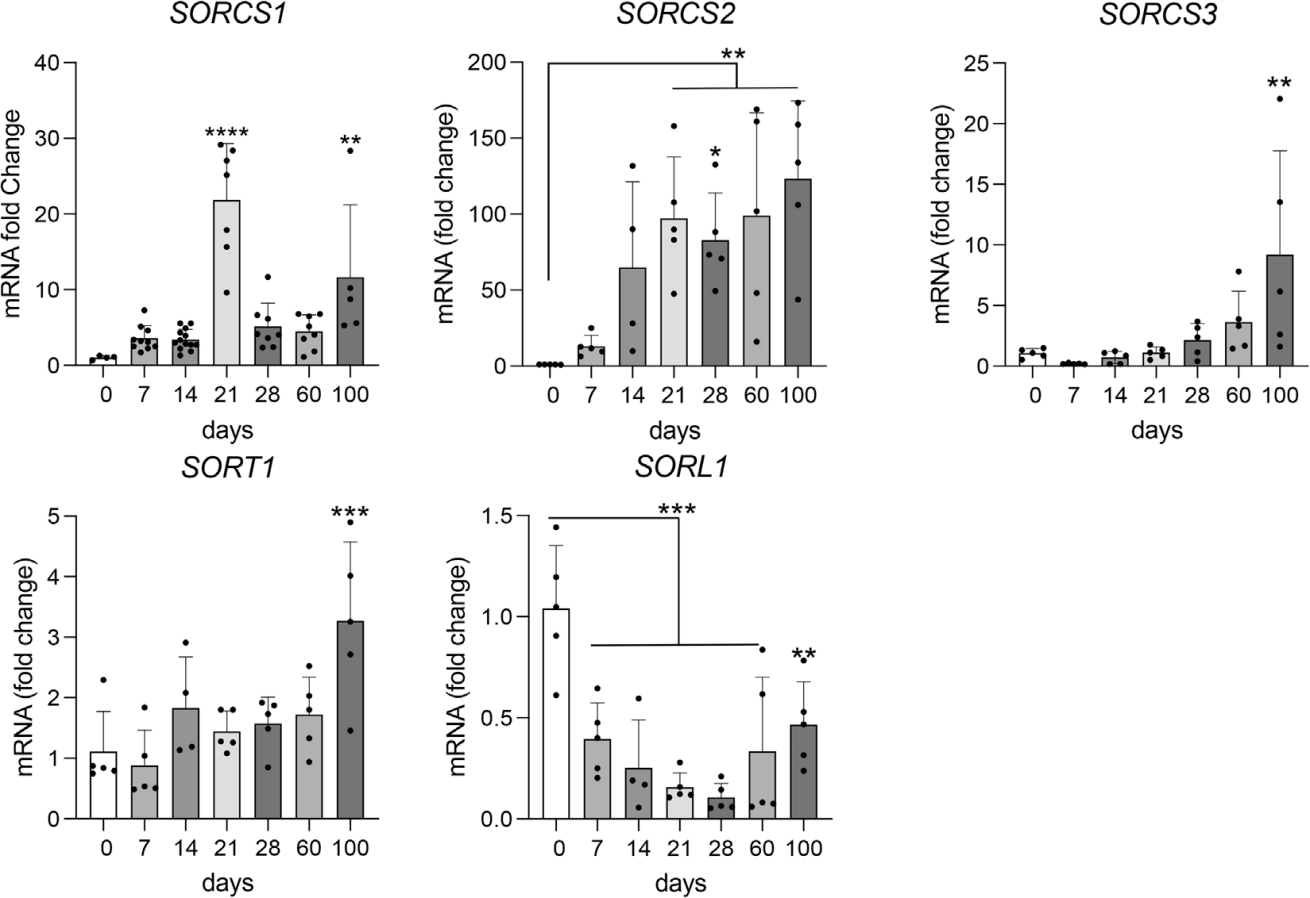
Transcript levels for VPS10P domain receptor genes during organoid maturation. Transcript levels for VPS10P domain receptor genes were determined by quantitative (q) RT-PCR in cerebral organoids at the indicated timepoints of differentiation. Data are given as changes relative to transcript levels in embryoid bodies (day 0 of differentiation set to 1). Statistical significance of data was determined by one-way ANOVA followed by Dunnett’s multiple comparisons tests. n = 4–13 organoids per condition (each organoid from an independent differentiation experiment); **, *p* < 0.01; ***, *p* < 0.001. ****, *p* < 0.0001.

In line with expression of VPS10P domain receptors in murine (https://www.informatics.jax.org) and human brains (https://www.proteinatlas.org), human cerebral organoids showed induction of receptor gene expression. However, distinct differences were noted comparing receptor species (Figure 4). Induction of gene transcription starting with early neurogenesis (day 14) was observed for *SORT1* (encoding sortilin) and *SORCS2*. A similar continuous increase in expression with organoid maturation was seen for *SORCS3*, although transcription was induced much later (from day 60) and remained overall low (Ct value 30.7 at day 100) as compared to *SORT1* (Ct value 23.1 at day 100) and *SORCS2* (Ct value 25.0 at day 100). By contrast, transcript levels for *SORL1* (encoding SORLA) were relatively high in EBs but continuously decreased with organoid formation (Ct value 27.1 at day 100). *SORCS1* transcripts showed a remarkable peak around day 21 and a subsequent decrease in levels in older organoids (Ct value 27.3 at day 100).

### Cerebral organoids express structurally distinct variants of VPS10P domain receptors

**FIGURE 5:**
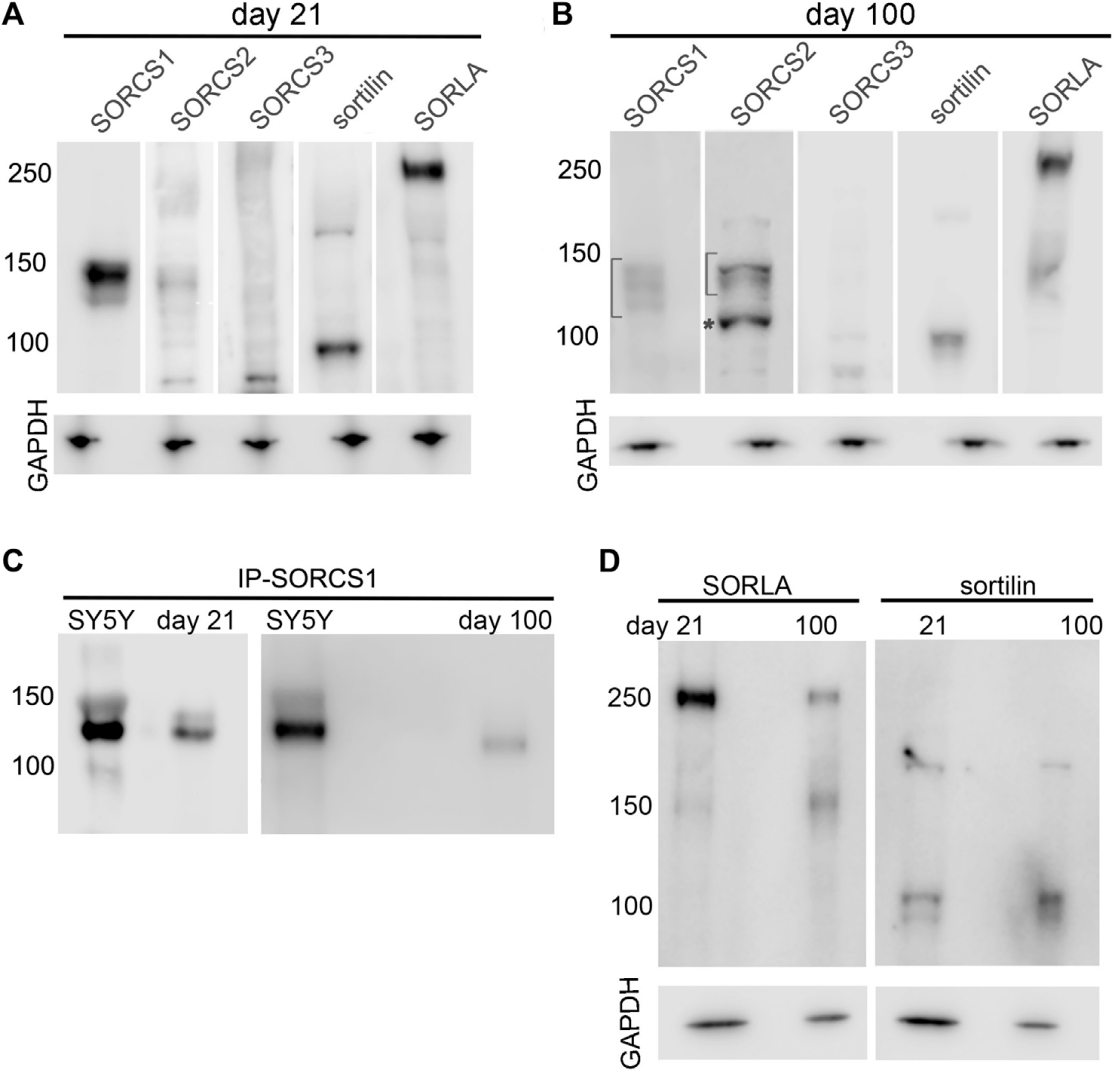
Expression of VPS10P domain receptors in cerebral organoids during maturation **(A,B)** Expression of VPS10P domain receptors was tested in membrane protein preparations from cerebral organoids at day 21 **(A)** and at day 100 **(B)** of differentiation using Western blotting. In B, brackets indicated the range of migration of various isoforms of SORCS1 and SORCS2 (130–150 kDa). The asterisk denotes the 104 kDa amino terminal fragment of the two-chain form of SORCS2 (104 kDa). Detection of GAPDH served as loading controls. **(C)** The soluble ectodomain of SORCS1 (approx. 120 kDa). was recovered by immunoprecipitation (IP-SORCS1) from soluble protein extracts of cerebral organoids at day 21 and day 100 of differentiation. The SORCS1 ectodomain recovered by IP from the cell supernatant of the neuroblastoma cell line SY5Y is shown as positive control. **(D)** Immunodetection of shedded ectodomains of sortilin (approx. 80 kDa) and SORLA (approx. 230 kDa) in soluble protein extracts from organoids at day 21 or day 100 of differentiation. For all panels, the migration of marker proteins of the indicated molecular weights in kDa are given.

While transcript levels constitute an accurate representation of receptor gene induction during cortical development, functional expression of VPS10P domain receptors is commonly regulated by post-transcriptional mechanisms, including glycosylation (Rovelet-Lecrux et al., 2021) or proteolytic processing into various receptor isoforms (Glerup et al., 2014). Therefore, we tested expression of VPS10P domain receptors in cerebral organoids at day 21 and day 100 of differentiation using Western blotting (Figure 5). Initial analyses were performed on organoid membrane preparations to detect full-length receptor variants inserted into cellular membranes (Figures 5A,B). Corroborating our qRT-PCR analyses, abundant levels of full-length SORCS1 protein (130 kDa) were seen in 21 days old organoids (Figure 5A). Also, sortilin (90 kDa), SORLA (250 kDa) and a faint band for SORCS2 (130 kDa) were detectable, whereas no discrete protein bands representing SORCS3 (130 kDa) were seen at this age (Figure 5A). At day 100 (Figure 5B), expression of all full-length receptors, with the exception of SORCS3, was apparent. In line with studies in mice and established cell lines (Hermey et al., 2003; Glerup et al., 2014), full-length SORCS1 and SORCS2 were present in several receptor isoforms as seen from multiple immunoreactive bands in the 130-150 kDa molecular weight range (brackets in Figure 5B). Also, detection of a 104 kDa protein band, representing the cleaved amino terminal domain of SORCS2 (asterisk in panel 5B), documented the presence of single- and two-chain variants of this receptor (compare Figure 1A for structure). These two SORCS2 variants have been reported in murine cell types and shown to carry distinct functions in trophic support of dopaminergic innervation (single-chain form) and in induction of cell death in Schwann cells (two-chain form) (Glerup et al., 2014). Overall, relative levels of SORCS1 variants decreased while levels of SORCS2 increased from day 21 to day 100, substantiating their converse temporal expression patterns.

As well as being expressed as full-length transmembrane receptors, several VPS10P domain receptor species are subject to proteolytic processing, releasing soluble fragments of the receptors’ ectodomains. Thus, we also performed Western blot analyses on post-membrane protein extracts to specifically detect soluble receptor fragments not associated with cell membranes (Figures 5C,D). Such soluble receptor fragments may originate from the extracellular or the cytosolic space of organoid tissues. In support of data from rodent cell models, SORCS1 in human cerebral organoids was undergoing proteolytic processing to release the soluble VPS10P domain of the receptor that acts as a diffusible factor in the circulation of mice facilitating insulin receptor signaling (Kjolby et al., 2020). This fact was documented by immunoprecipitation of the SORCS1 ectodomain from soluble protein extracts of organoids at day 21 and day 100 (Figure 5C). As to be expected from its temporal expression patterns, levels of soluble SORCS1 were more abundant at day 21 than at day 100 of organoid differentiation. In addition to SORCS1, soluble ectodomains are also produced from SORLA and sortilin by membrane shedding (Hampe et al., 2000; Hermey et al., 2006). These receptor fragments were also detectable in soluble protein extracts at day 21 and day 100 (Figure 5D). Although the functional relevance of these receptor fragments remains poorly explained, they bear significance as surrogate biomarkers in blood or cerebrospinal fluid, presumably mirroring levels of receptor expression in tissues (Ma et al., 2009; Carrasquillo et al., 2010). In support of these earlier hypotheses, levels of soluble SORLA decreased from day 21 to day 100, while levels of soluble sortilin increased during this time, reflecting the observed changes in gene transcription.

### VSPS10P domain receptors show spatially distinct patterns of expression in neuronal and non-neuronal brain cell types

**FIGURE 6:**
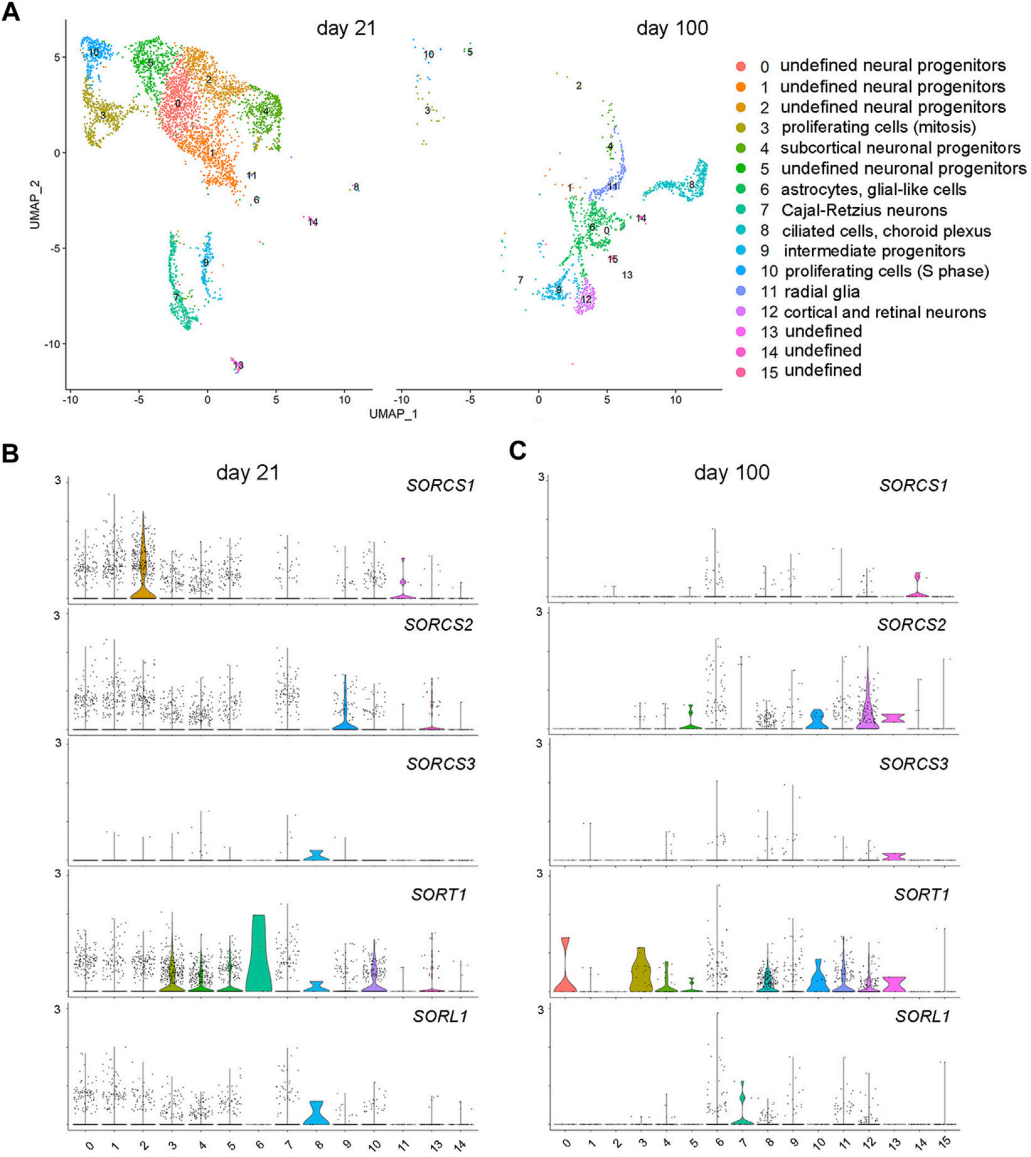
Brain cell-type specific expression of VPS10P domain receptors identified by single-cell RNAseq. **(A)** Combined single-cell transcript datasets from 21 to 100 days old cerebral organoids were analyzed by unsupervised clustering and visualized using Uniform Manifold Approximation and Projection for dimension reduction (UMAP) plot. A total of 16 distinct cell clusters were identified. **(B–C)** Violine plots localizing VPS10P domain receptor transcripts to the indicated clusters in organoids at day 21 **(B)** and day 100 **(C)** of differentiation.

To explore the cell-type specific expression of VPS10P domain receptors in cerebral organoids, we performed single-cell RNA sequencing (scRNAseq) in organoids at day 21 and day 100 of differentiation. Day 21 was chosen for early organoid development as it represented the time of peak expression of *SORCS1*. While scRNAseq of early organoids pose few technical challenges, analysis of mature organoids tends to be more difficult, due to their larger size and their necrotic core, hampering the generation of high-quality single-cell suspensions. To address these technical obstacles, we developed novel protocols to generate single-cell suspensions from 100 days old organoids with a reproducible cell viability of approximately 80% (see methods for details). Analysis of the combined single-cell transcript datasets of 21 days and 100 days old organoids by unsupervised clustering, and visualized using Uniform Manifold Approximation and Projection for dimension reduction (UMAP) plot, identified 16 distinct cell clusters (Figure 6A; Supplementary Figure S1). Recapitulating brain development, 21 days old organoids were characterized by large clusters of neural progenitors (clusters 0, 1, 2) as well as neuronal and subcortical neuronal progenitors (clusters 4 and 5). Also, clusters of proliferating cells in S-phase (cluster 10) or mitosis (clusters 3), as well as intermediate progenitors (cluster 9) and Cajal-Retzius neurons (cluster 7) were identified at this stage. In mature organoids (day 100), clusters of various progenitors and proliferating cells had largely disappeared. Instead, cell types indicative of further cortical specification were evident, including intermediate progenitors (cluster 9) as well as cortical and retinal neurons (cluster 12). Radial glia (cluster 11), astrocytes (cluster 6), and ciliated cells of the choroid plexus (cluster 8) were non-neuronal cell types identified at day 100.

Corroborating distinct temporal patterns of receptor gene expression seen by qRT-PCR, *SORCS1* transcripts were abundant in 21 days old organoids and exclusively localized to one class of neural progenitor (cluster 2; Figure 6B). Receptor transcripts were significantly reduced in organoids at day 100, in line with loss of this neural progenitor population during maturation (Figure 6C). Induction of *SORCS2* transcription with organoid maturation was also recapitulated in the scRNAseq dataset as abundance of the transcript increased from day 21 (in intermediate progenitor cluster 9) to day 100 (in mature cortical neuron cluster 12) (Figures 6B,C). *SORT1* showed the most wide-spread expression in early and mature organoids including in proliferating cells (clusters 3 and 10), neuronal progenitors (clusters 4 and 5), and mature cortical and retinal neurons (clusters 12), but also in radial glia (cluster 11), astrocytes (cluster 6), and ciliated cell types (cluster 8) (Figures 6B,C). By contrast, transcript levels for *SORL1* and *SORCS3* were generally low, both at day 21 and day 100 (Figures 6B,C). These findings substantiated predominant *SORL1* gene transcription in EBs, and a general absence of *SORCS3* transcripts from cerebral organoids at any age. Of note, the inherent lack of microglia in cerebral organoids precluded us from substantiating the expression of VPS10P domain receptors, notably *SORL1*, in this cell type. However, we have reported *SORL1* expression in iPSC-derived human microglia using specialized differentiation protocols before (Kaminska et al., 2023).

**FIGURE 7:**
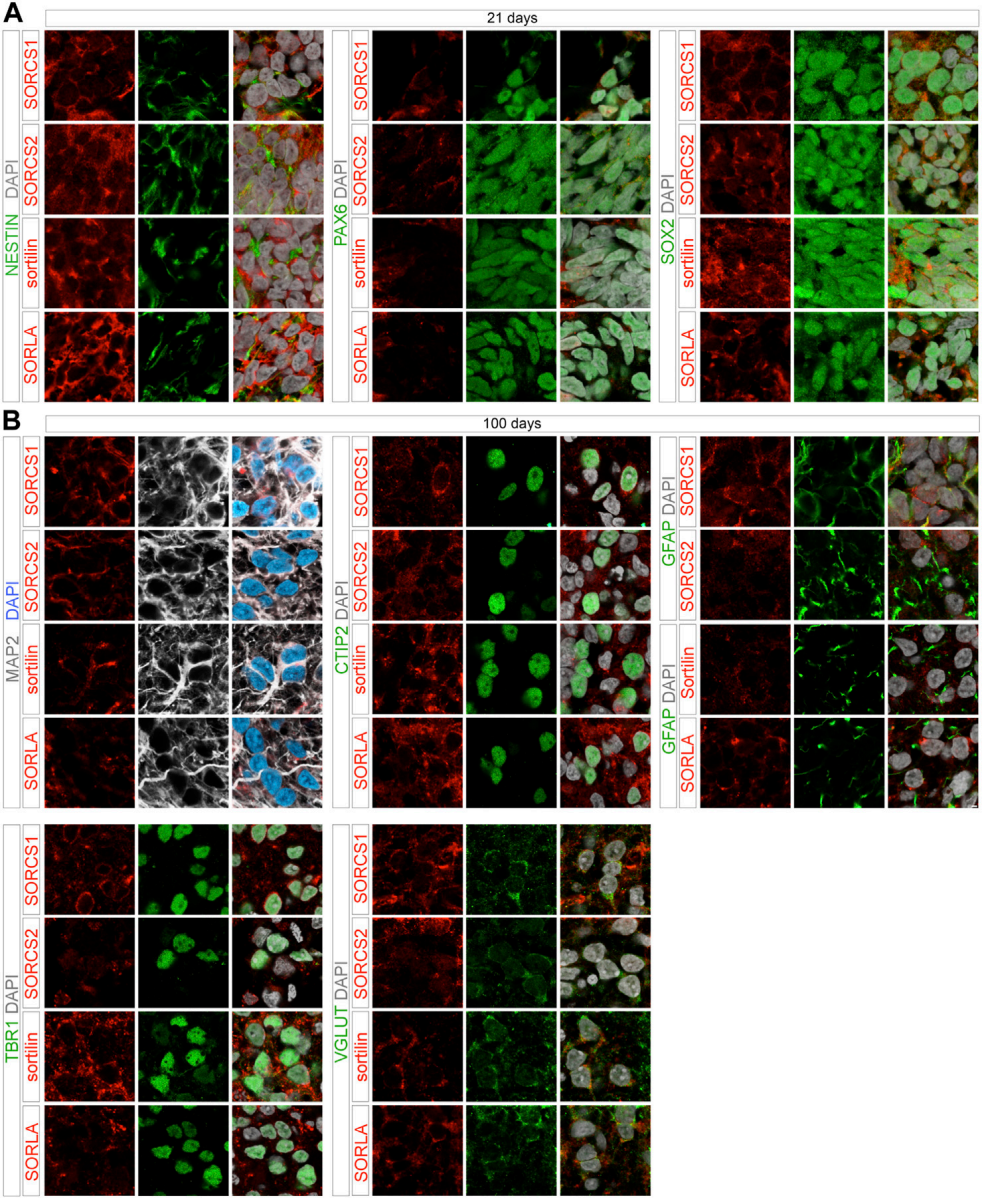
Cell-type specific expression of VPS10P domain receptors in 21 and 100 days old organoids. Immunohistological detection of the indicated VPS10P domain receptors in 21 days **(A)** or 100 days **(B)** old cerebral organoids. Cell-type specific expression of the receptors (red) was tested by colocalization with the indicated cell markers (green, grey). Single (left, middle) as well as merged channel configurations with DAPI counterstain (right) are shown. Scale bars: 2 μm.

Cell-type specific expression of the various receptors was supported by immunohistochemical analyses of cerebral organoids. At day 21, SORCS1, SORCS2, sortilin, as well as SORLA were all seen in neural progenitors characterized by expression of transcription factors SOX2 and PAX6 as well as by intermediate filament protein nestin, established markers of early telencephalic development. At day 100, robust levels of SORCS1, SORCS2, and sortilin were detected in cortical neurons, characterized by expressing of the microtubule-associated protein MAP2, transcription factors TBR1 and CTIP2, as well as vesicular glutamate transporter VGLUT (Figure 7B). Despite a substantial drop in transcript levels in organoids as compared to EBs, SORLA protein was also clearly detectable in cortical neurons at day 100 of maturation (Figure 7B). Based on these data, expression of all four receptors in distinct progenitor populations during development and in cortical neurons in the mature human brain is a valid assumption. In addition to neuronal cell types, SORLA, sortilin, and SORCS1 were also expressed in GFAP-positive astrocytes in 100 days old organoids (Figure 7B). Lower astrocytic expression was seen for SORCS2 (Figure 7B), supporting findings from mouse models that expression of this receptor in astrocytes is only induced during stress response, as in stroke (Malik et al., 2020).

### Subcellular localization supports distinct roles for VSPS10P domain receptors in intraneuronal protein transport

VPS10P domain receptors act as sorting factors that move target proteins to their destined location within cells. To perform this task, the receptors, and their bound cargo, follow a complex trafficking path between Golgi, cell surface, and endocytic compartments (Malik and Willnow, 2020). To interrogate distinct subcellular localization patterns in human neurons, we co-immunostained VPS10P domain receptors with markers of various intracellular compartments in 21 days and 100 days old organoids. Because of a general absence of SORCS3 from cerebral organoids, we focused our analyses on SORCS1, SORCS2, SORLA, and sortilin.

**FIGURE 8:**
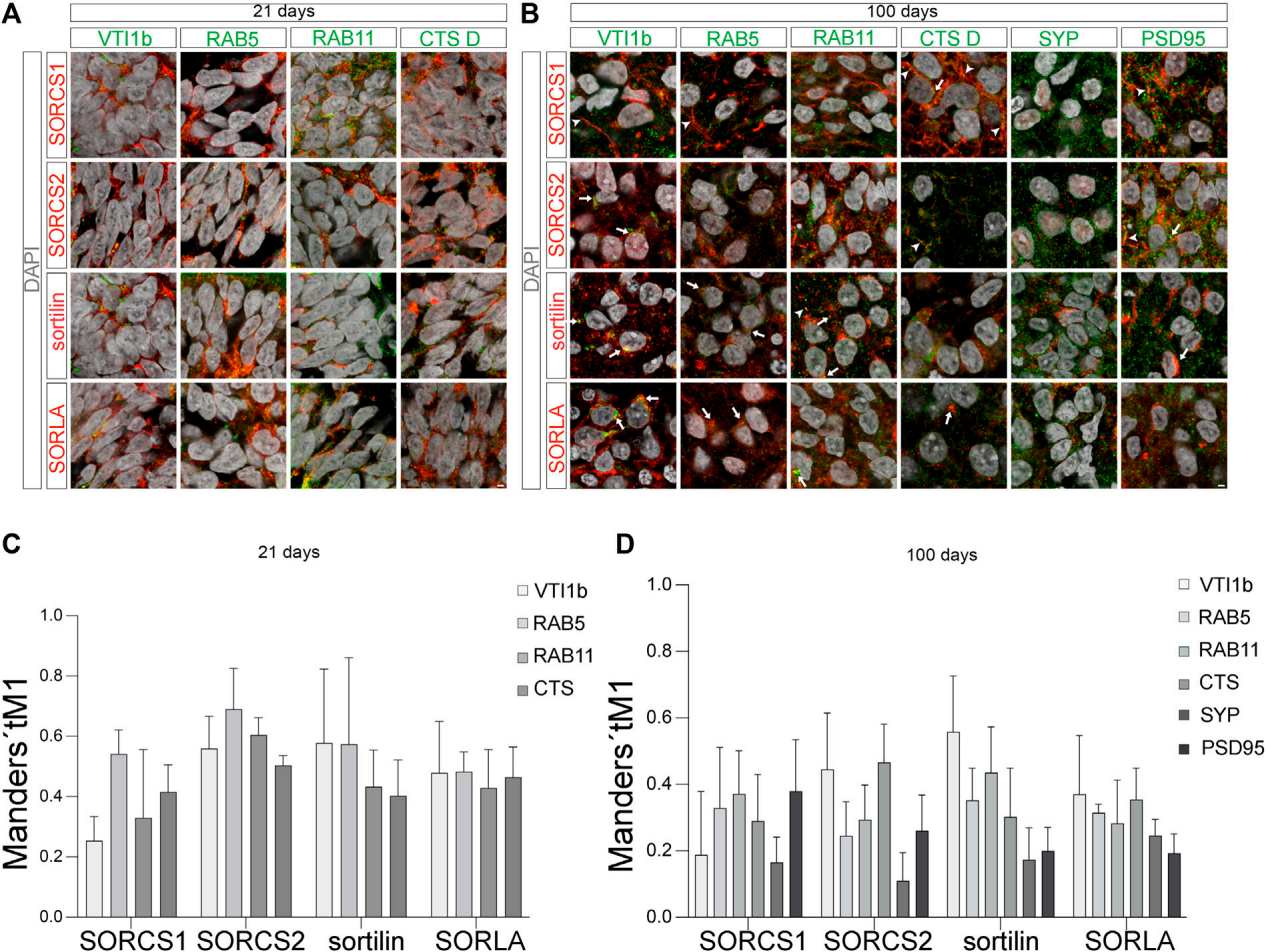
Subcellular localization of VPS10P domain receptors in 21 days and 100 days old organoids **(A,B)** Colocalization of SORCS1, SORCS2, sortilin, and SORLA (red) with the indicated organelle markers (green) in neural progenitors [**(A)**, day 21] and in cortical neurons [**(B)**, day 100] was tested using immunohistochemistry. Merged channel configurations include nuclear counterstain with DAPI (grey). Arrowheads in B mark the localization of individual receptors to neuronal projections in exemplary images. Arrows in B highlight co-localization of the receptors with the given markers. Scale bars: 2 μm. **(C,D)** The extend of colocalization of VPS10P domain receptors with the indicated cell markers [as exemplified in **(A,B)**] was quantified using Mander’s coefficient tM1 [**(C)**, day 21; **(D)**, day 100]. Data were collected from 20–40 cells of 4 image per condition and are given as mean ± SD.

At day 21, all four receptors we showed a comparable subcellular distribution in neural progenitors that was consistent with a sorting path between cell surface, endosomal compartments, and the *trans*Golgi network (TGN) (Figures 8A,C). In detail, the receptors colocalized with TGN marker VTI1B, with RAB5 and RAB11, markers of early and recycling endosomes, respectively, as well as with cathepsin D (CTS D), indicative of lysosomes. This pattern is characteristic of endocytosis with receptors sorting internalized cargo from early endosomes (RAB5) to lysosomal compartments (cathepsin D), and returning back to the cell surface via recycling endosomes (RAB11). For example, this route is taken by sortilin when internalizing progranulin or apolipoprotein E, factors implicated in FTD and AD, respectively (Hu et al., 2010; Carlo et al., 2013). Additionally, localization of the receptors to the TGN (VTI1B) recapitulated an alternative route whereby these receptors sort some internalized proteins from early endosomes back to the Golgi to circumvent lysosomal catabolism. Exemplary, this is the path whereby SORLA sorts APP, the etiologic agent in AD (Andersen et al., 2005; Dumanis et al., 2015). Obviously, immunosignals for the receptors seen in endocytic compartments may represent full-length receptors but possibly also soluble receptor fragments internalized from the extracellular space. In particular, this situation may apply to (soluble) SORCS1 during brain development, as immunosignals for this receptor were much more abundant in early endosomes (clearance of cargo) than in recycling endosomes (cell surface sorting of full-length receptors) (Figure 8C).

While the relative distribution of VPS10P domain receptors between the tested intracellular compartments was comparable in early progenitors, the situation was different in cortical neurons at day 100 of organoid differentiation (Figures 8B,D). While SORLA mainly localized to the perinuclear region and intracellular vesicles in the soma, strong SORCS1 immunoreactivity was seen in the soma but also in neuronal projections (arrowheads in Figure 8B). Like SORLA, sortilin mainly localized to the perinuclear region, however some immunofluorescence signal was also present in neuronal projections (arrowheads in Figure 8B). SORCS2 showed a distinct punctate pattern both in somatic vesicles and in neuronal projections (Figure 8B). Co-immunostainings with Golgi marker VTI1B, endosomal markers RAB5 and RAB11, lysosomal marker cathepsin D, as well as with synaptophysin (SYP) and PSD95, indicative of pre- and post-synaptic compartments, refined analysis of subcellular receptor distributions. In line with predominant localization to the soma, relative abundance of SORLA and sortilin was highest in the TGN, followed by localization to the endo-lysosomal system (Figure 8D). Abundance at the pre- (SYP) or post-synapse (PSD95) was relatively lower, with no apparent preference for either synaptic compartment. Overall, these patterns supported established roles for both receptors in endocytosis as well as TGN to endo-lysosomal sorting in cortical neurons. In addition to TGN and endosomal markers, SORCS1 showed significant co-localization with PSD95 but not SYP, indicating its localization to the post-synaptic compartment (Figure 8D). Similar to SORCS1, SORCS2 localized to TGN and endosomes, but also to the post-synaptic compartments. These findings agreed with sorting paths for both receptors in the soma but also along neuronal projections and at the post-synapse.

## Discussion

In this study, we applied cerebral organoid models to interrogate the expression of VPS10P domain receptors during neural development and differentiation. Our studies uncovered differences in temporal, spatial, and subcellular expression patterns for the various receptor species that argue for divergent roles in development and adult brain function, and for distinct modes of action in sorting paths in somatodentritic compartments of human neurons. As a caveat, our analyses are based on studies in a single iPSC line. However, this line has been subjected to in-depth quality control by the provider and by us, and it has been validated by use in numerous publications by others (https://hpscreg.eu/cell-line/HMGUi001-A).

While the importance of VPS10P domain receptors as major risk genes in neurodevelopmental and neurodegenerative diseases is well recognized, their mode of action in various disease contexts remains far from being well understood. Conceptualizing distinct molecular functions for various receptor species is complicated by their close structural resemblance, in particular seen for SORCS1, -2 and -3. Also, the ability of the receptors to interact with a multitude of ligands, some of which being shared by all receptors (e.g., tropomyosin receptor kinase (Trk) receptor B), complicates functional distinction (Nykjaer and Willnow, 2002; Malik and Willnow, 2020). Based on work in this study, differences in spatial and temporal expression of the receptors in human brain tissues likely explain some of their (patho) physiological traits.

Obvious distinctions of VP10P domain receptors are seen in their temporal patterns of expression. As deduced from qRT-PCR and scRNASeq analyses, *SORL1* represents a marker of early stem cell fate while *SORCS1* acts in progenitors at a time of cortical induction. *SORT1* and *SORCS2* constitute markers of more mature human brain cell types. Low levels of *SORCS3* transcripts recapitulate the predicted insignificant expression of the receptor in human cortical tissue (https://www.proteinatlas.org). By and large, temporal patterns of gene transcription are corroborated by detection of the receptor proteins in early and mature organoids in this study, arguing for a prominent transcriptional control of receptor functions. However, some differences are noteworthy that highlight the importance of post-transcriptional mechanisms in defining receptor activities. In particular, this interpretation concerns the AD risk factor SORLA. Recent scRNAseq analyses suggested that its expression in the human brain may be restricted to microglia (Gosselin et al., 2017; Hansen et al., 2018). However, although transcript levels progressively decrease with differentiation from EB to mature organoids, robust levels of the protein can be detected in cortical neurons at day 100 of organoid differentiation. These finding supports earlier studies of immunohistological analyses in human brain specimens (Scherzer et al., 2004; Offe et al., 2006) and clearly support a neuronal role for this receptor. As well as protein stability, proteolytic processing also emerges as possible post-transcriptional mechanism to define receptor functions. Based on prior studies in established cell lines and mouse models (Hermey et al., 2003; Glerup et al., 2014), the detection of soluble forms of SORCS1 and of a two-chain variant of SORCS2 in human cerebral organoids in this study supports the relevance of these processing products for the human brain as well.

VPS10P domain receptors are largely recognize for their role in intracellular protein trafficking between cell surface and secretory or endocytic compartments. Receptor routing is governed cytosolic adaptors, such as retromer, GGAs, and PACS1, that interact with binding motifs in the receptor tails (Nielsen et al., 2001; Fjorback et al., 2012; Dumanis et al., 2015). Receptor-mediated sorting of cargo has been studied in multiple mammalian cell types, but protein sorting in soma and along neurite extensions of neurons has received major attention. Typically, the bulk of the receptor mass localizes to the TGN, the major hub for sorting of proteins between endocytic and secretory compartments. Yet, differences in the composition of adaptor binding motifs in the tails of various VPS10P domain receptors, and the modulation of tail binding sites by alternative splicing (Hermey et al., 2003; Nielsen et al., 2008), argue for different sorting pathways for individual receptors. This assumption is supported by our immunohistological analyses of receptor distribution in cortical neurons that suggest prominent roles for SORLA and sortilin in neuronal endocytosis and/or endo-lysosomal sorting of proteins. With relevance to brain disease, SORLA acts as a sorting receptor for APP, moving internalized APP from endosomes retrogradely to the TGN to prevent its breakdown into amyloid-β peptides in early endosomes (Andersen et al., 2005; Burgert et al., 2013; Dumanis et al., 2015). Contrasting SORLA and sortilin, localization of SORCS1 and SORCS2 to the soma about also to post-synaptic compartments suggest specific roles for these two receptors in protein sorting along neurite tracks. In line with this hypothesis, SORCS1 and -2 have been shown to sort the neurotrophin receptor TrkB to synaptosomal membranes, controlling neurotrophic signal reception in target cells (Glerup et al., 2016; Subkhangulova et al., 2018).

## Data Availability

All data are available on reasonable request from the authors. The scRNAseq data are available from the GEO database (accession numbers GSE240153 for day 21, and GSE233567 for day 100).
