# Supplementary material for: Spatially and temporally distinct patterns of expression for VPS10P domain receptors in human cerebral organoids
Source: Front Cell Dev Biol. 2023 Sep 29;11:1229584. doi: 10.3389/fcell.2023.1229584 (PMC10570844; doi:10.3389/fcell.2023.1229584)
Supplement: Supplementary file 1 [file Table1.docx]

**SUPPLEMENTARY TABLES**

**Supplementary table 1: Taqman probes used for quantitative RT-PCR**

| **Genes** | **Manufacturer** | **Catalog No.** | **Species** |
| --- | --- | --- | --- |
| *CTIP2 (BCL11B)* | Thermo Scientifics | Hs01102259_m1 | Human |
| *FOXG-1* | Thermo Scientifics | Hs01850784_s1 | Human |
| *GAPDH* | Thermo Scientifics | Hs99999905_m1 | Human |
| *GFAP* | Thermo Scientifics | Hs00909233_m1 | Human |
| *MAP2* | Thermo Scientifics | Hs00258900_m1 | Human |
| *NANOG* | Thermo Scientifics | Hs02387400_g1 | Human |
| *OLIG2* | Thermo Scientifics | Hs00300164_s1 | Human |
| *OTX2* | Thermo Scientifics | Hs00222238_m1 | Human |
| *PAX6* | Thermo Scientifics | Hs00242217_m1 | Human |
| *POU5F1 (OCT4)* | Thermo Scientifics | Hs00999632_g1 | Human |
| *S100B* | Thermo Scientifics | Hs00902901_m1 | Human |
| *SorCS1* | Thermo Scientifics | Hs00364666_m1 | Human |
| *SorCS2* | Thermo Scientifics | Hs01019137_m1 | Human |
| *SorCS3* | Thermo Scientifics | Hs01039447_m1 | Human |
| *SORL* | Thermo Scientifics | Hs00983772_m1 | Human |
| *SORT1* | Thermo Scientifics | Hs00361760_m1 | Human |
| *SOX2* | Thermo Scientifics | Hs01053049_s1 | Human |
| *TBR-1* | Thermo Scientifics | Hs00232429_m1 | Human |
| *TBR-2 (EOMES)* | Thermo Scientifics | Hs00172872_m1 | Human |
| *TUBB3* | Thermo Scientifics | Hs00964963_g1 | Human |
| *VGLUT2 (SLC17A6)* | Thermo Scientifics | Hs00220439_m1 | Human |

**Supplementary table 2: Primary antibodies used in this study**

Amino acid numbers of the indicated antigen epitopes refer to the human receptor sequences. IF, immunofluorescence; IP, immunoprecipitation; WB, western blotting.

| **Antibody** | **Manufacturer** | **Catalog No.** | **Host** | **Dilution** |
| --- | --- | --- | --- | --- |
| β−actin | Abcam | AB8227 | Rabbit | 1:1000 |
| Cathepsin D | R&D System | MAB10141 | Mouse | 1:500 |
| CTIP2 | Abcam | AB18465 | Rat | 1:500 |
| NESTIN | ABCAM | AB105389 | Rabbit | 1:200 |
| EEA1 | BD Biosciences | 610457 | Mouse | 1:200 |
| FOXG1 | Abcam | AB227888 | Rabbit | 1:100 |
| GAPDH | Genetex | GTX627408-01 | Mouse | 1:2000 |
| GFAP | Abcam | AB53554 | Goat | 1:200 |
| Ki67/MKI67 | R&D System | AF7617 | Sheep | 1:40 |
| MAP2 | Synaptic System | 188004 | Guinea pig | 1:1000 |
| OLIG4-PE | Melteny | 130-109-199 | Mouse | 1:10 |
| PAX6 | Biolegend | 901301 | Rabbit | 1:400 |
| PSD 95 | Abcam | AB2723 | Mouse | 1:200 |
| RAB5A | Synaptic System | 108011 | Mouse | 1:500 |
| RAB11 | Cell Signaling | 5589 | Rabbit | 1:50 |
| SORCS1 (E996-T1095) | Sigma | HPA011948 | Rabbit | 1:250  (4µl for IP) |
| SORCS1 (S111-S1099) | R&D System | AF3457 | Goat | 1:70 (IF) 1:1000 (WB) |
| SORCS2 (R51-G1079) | DAKO F7100 |  | Rabbit | 1:200 (IF)  1:1000 (WB) |
| SORCS2 (S70-G1078) | R&D System | AF4238 | Sheep | 1:40 (IF) |
| SORCS3 (E34-S1122) | R&D System | MAB30671 | Mouse | 1:1000 (WB) |
| SORLA | BD Bioscience | 611861 | Mouse | 1:1000 (WB) 1:1000 (IF) |
| SORLA (S124-V2136) | in house | Mouse | Goat | 1:40 |
| Sortilin | BD bioscience | BD612101 | Mouse | 1:1000 (WB) |
| Sortilin (S78-N755) | R&D | AF3154 | Goat | 1:200 |
| SOX2 | R&D System | MAB2018 | Mouse | 1:500 |
| Synaptophysin | ENZO LIFE | ADI-905-782 | Mouse | 1:1000 |
| S100β | Dako | GA504 | Rabbit | 1:200 |
| TBR1 | Abcam | AB31940 | Rabbit | 1:200 |
| β-tubulin | Merk | MAB1637 | Mouse | 1:1000 |
| VGLUT | Synaptic System | 135403 | Rabbit | 1:500 |
| VTi1b | BD Biosciences | 611405 | Mouse | 1:150 |
| DAPI | Invitrogen | D1306 |  | 1:1000 |

**Supplementary table 3: Secondary antibodies used in this study**

| **Antibody** | **Manufacturer** | **Catalog No.** | **Host** | **Dilution** |
| --- | --- | --- | --- | --- |
| anti-mouse Alexa Fluor 488 | Invitrogen | A21202 | Donkey | 1:1000 |
| anti-mouse Alexa Fluor 568 | Invitrogen | A10037 | Donkey | 1:1000 |
| anti-mouse Alexa Fluor 647 | Invitrogen | A31571 | Donkey | 1:1000 |
| anti-goat Alexa Fluor 568 | Invitrogen | A11057 | Donkey | 1:1000 |
| anti-goat Alexa Fluor 568 | Invitrogen | A21447 | Donkey | 1:1000 |
| anti-rabbit Alexa Fluor 488 | Invitrogen | A21206 | Donkey | 1:1000 |
| anti-rabbit Alexa Fluor 568 | Invitrogen | A10042 | Donkey | 1:1000 |
| anti-rabbit Alexa Fluor 647 | Invitrogen | A31573 | Donkey | 1:1000 |
| anti-guinea pig Alexa Fluor 647 | Jackson Immuno | 706-605-158 | Donkey | 1:200 |
