# Supplementary figures and images for: Spatially and temporally distinct patterns of expression for VPS10P domain receptors in human cerebral organoids
Source: Front Cell Dev Biol. 2023 Sep 29;11:1229584. doi: 10.3389/fcell.2023.1229584 (PMC10570844; doi:10.3389/fcell.2023.1229584)

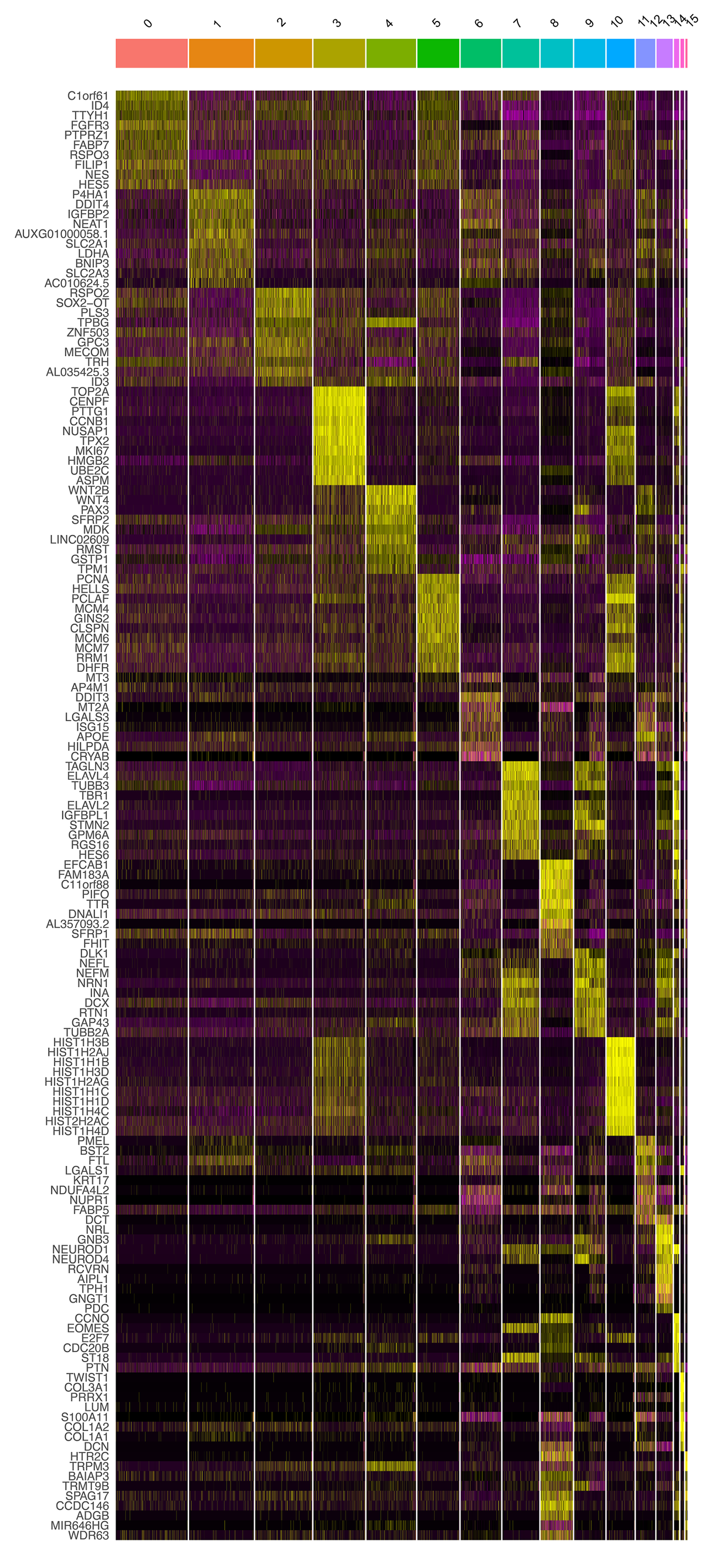

Supplement: Supplementary file 2 [file Image1.TIF]
