# Supplementary material for: Spatially and temporally distinct patterns of expression for VPS10P domain receptors in human cerebral organoids
Source: Front Cell Dev Biol. 2023 Sep 29;11:1229584. doi: 10.3389/fcell.2023.1229584 (PMC10570844; doi:10.3389/fcell.2023.1229584)
Supplement: Supplementary file 3 [file Table2.DOCX]

**SUPPLEMENTARY FIGURE**

**Supplementary figure 1:**  Heatmap showing the top ten most unique identifiers per cluster in the combined day 21 and day 100 single-cell RNAseq data set. Genes were identified using the Seurat function "FindAllMarkers" and visualized using the Seurat function "DoHeatmap".
